# Chinese Herbal Medicine for Osteoporosis: A Systematic Review of Randomized Controlled Trails

**DOI:** 10.1155/2013/356260

**Published:** 2013-01-30

**Authors:** Zhi-qian Wang, Jin-long Li, Yue-li Sun, Min Yao, Jie Gao, Zhu Yang, Qi Shi, Xue-jun Cui, Yong-jun Wang

**Affiliations:** Longhua Hospital, Shanghai University of Traditional Chinese Medicine, 725 South Wanping Road, Shanghai 200032, China

## Abstract

*Background*. Osteoporosis is a major health problem for the elderly population. Chinese herb may be beneficial to osteoporosis due to its capability. *Objectives*. This study was designed to evaluate the effectiveness of Chinese medicine treatment on the patients with osteoporosis. *Search Methods*. Randomized controlled trials were retrieved from different 9 databases. *Results*. This meta analysis included 12 RCTs involving 1816 patients to compare Chinese herbs with placebo or standard anti-osteoporotic therapy in the treatment of bone loss. The pooled data showed that the percent change of increased BMD in the spine is higher with Chinese herb compared to placebo (lumber spine: WMD = 0.07, 95% CI: 0.01–0.04). In the femoral, Chinese herb showed significantly higher increments of BMD compared to placebo (femoral neck: WMD = 0.06, 95% CI: −0.02–0.13). Compared to the other standard anti-osteoporotic drugs, Chinese herbs also show advantage in BMD change (lumber spine: WMD = 0.03, 95% CI: −0.01–0.08; femoral: WMD = 0.01, 95% CI: −0.01–0.02). *Conclusions*. Our results demonstrated that Chinese herb significantly increased lumbar spine BMD as compared to the placebo or other standard anti-osteoporotic drugs.

## 1. Introduction 

Osteoporosis, the thinning of bone due to the net loss of calcium and bone structure, occurs primarily with ageing. It results in a great cost both to the individual suffering from bone fractures and society burdened with the extreme financial costs [1]. In the last decade, pharmaceutical companies developed a rich collection of new drugs that offer specific and clearly targeted therapeutic effects on the improvement of bone quality. The new drugs have been developed based on the understanding of the metabolism of the bone tissue so that building it up could work through the stimulation of the anabolic side or suppression of the catabolic side. Teriparatide and strontium products represent the former and the bisphosphonates the latter [2, 3]. While therapeutic drugs created for the treatment of osteoporosis still show some side effects. Firstly, because the therapeutic agents offer an unbalanced effect through a specific artificial influence on the end of the metabolic cycle of bone physiology, the structural changes of bones from long-term treatment remains unknown. Secondly, adverse reactions of the therapeutic agents may yet appear minor; uncertainty exists with longer uses [4, 5]. Indeed, odd fractures were already reported with prolonged treatment, and large doses of bisphosphonates could induce osteonecrosis of the jaw bone.

 Medical scientists in China have studied many medicinal herbs and found that they have antiosteoporotic effects in the laboratory and subsequently in clinical trials [6]. But its effects on bone metabolism and calcium homeostasis are still not clear. During the last 10 years, some unique clinical trials focused on Chinese herbs in the treatment of osteoporosis have been conducted. 

 It would be valuable to evaluate the quality of these trials and assess the efficacy and safety data provided by the trials in terms of the principles and measurements of evidence-based medicine. In this study, a systematic review and meta-analysis of randomized controlled trail (RCTs) were performed to compare Chinese herbs with standard anti-osteoporotic drugs or placebo and to identify herbs commonly used in the clinical treatment of bone loss. We hypothesized that the eligible trials would provide evidence of the effect of Chinese herbs on bone mineral density (BMD) and the therapeutic benefits of Chinese medicine treatment in patients with bone loss.

## 2. Materials and Methods

### 2.1. Eligibility Criteria

All RCTs comparing the efficacy of Chinese herbs for the treatment of osteoporosis were included.

 The diagnostic criteria for osteoporosis or osteopenia in the trials were required to be in accordance with the criteria of the World Health Organization (WHO 1994) [7]. Participant selection excluded those receiving any medications known to affect bone or calcium metabolism, including current use or a history of 3-month (or more) use of exogenous estrogens, corticosteroids, and thiazine; those with any systemic, endocrine disease, or any surgery known to affect bone health (i.e., ovariectomy, excision of thyroid gland or intestinal tract, etc.), as well as cancer.

 The intervention was required to be oral administration of any kind of herbal preparation used alone or in combination with other herbs for subjects in treatment groups and placebo or other standard anti-osteoporotic drugs for subjects in the control groups. Concurrent administration of calcium and/or vitamin D was acceptable if both groups received the same dose and formulation.

 The outcome measures included changes in lumbar, femoral, and forearm BMD values and also the followup of the patients must be at least for six months.

### 2.2. Search Methods for Identification of Studies

A literature search was performed using the phrase “chinese herb AND herbal AND osteoporosis AND fracture” with the limits “randomized controlled trail” and regardless of language or publication status.

 A total of 9 electronic databases were searched including MEDLINE (1966 to February 2012), EMbase (1974 to February 2012), Chinese Biomedical Literature Database (CBM, 1978 to February 2012), China National Knowledge Infrastructure (CNKI, 1994 to February 2012), the Chinese Scientific and Technical Journals database (VIP, 1989 to February 2012), Wanfang Data (1995 to February 2012), China Doctoral Dissertations Full-text Database (CDFD, 1984 to February 2012), China Master's Theses Full-text Database (CMFD, 1984 to February 2012), and China Proceedings of Conference Full-text Database (CPFD, 2000 to February 2012). 

 At the same time, we searched references of the included studies for any possible titles matching the inclusion criteria. We also wrote emails to the author to request for the data of the trials which did not report the original data.

### 2.3. Selection of Studies

Two reviewers independently selected articles. The titles and abstracts of articles found in the search were screened by ZQW and JLL, who discarded trials that were clearly not eligible. Full article was selected by two review authors independently (ZQW and JLL). 

### 2.4. Assessments Bias Risk

 Risk of bias was assessed independently by two review authors (Z.-Q. Wang and JLL) with the criteria in the Cochrane Handbook for Systematic Reviews of Interventions 5.1.0 [8]. Sequence generation, allocation concealment, blinding (or masking), incomplete data assessment, selective outcome reporting, and other sources of bias were assessed with three potential responses: yes, no, and unclear. Disagreements between review authors were resolved by discussion or with a third author (X.-J. Cui).

### 2.5. Data Extraction and Management

Information was carefully extracted from all eligible publications independently by two of the authors of the present study (Z.-Q. Wang and WZQ). Two review authors (Y.-L. Sun and X.-J. Cui) checked and entered data into Review Manager (RevMan 5.1). We two graded the methodology quality of all included trails by JADAD [9] (Table 1) and “Risk of Bias table” (Table 2) which recommended by Cochrane handbook 5.0. Risk of bias was assessed independently by two review authors (Z.-Q. Wang and J.-L. Li) with the criteria in the Cochrane Handbook for Systematic Reviews of Interventions 5.1.0 [8]. Sequence generation, allocation concealment, blinding (or masking), incomplete data assessment, selective outcome reporting, and other sources of bias were assessed with three potential responses: yes, no, and unclear. Disagreements between review authors were resolved by discussion or with a third author (X.-J. Cui). The data extracted consisted of number of patients with lumbar spine and hip areal BMD during follow-up. 

### 2.6. Measures of Treatment Effect

Statistical analysis was performed with RevMan 5.1 software. We expressed continuous data as weighted mean differences (WMD) with 95% CI, or as standardized weighted mean differences (SMD) if outcomes were conceptually the same but measured in different ways in the different trials. Statistical heterogeneity was investigated using the x2 test and I2 statistic (I2 represents the percentage of variability due to between-study variability). We tested heterogeneity among trial results using the I2 statistic. We considered a value greater than 50% as substantial heterogeneity. We calculated an estimate of the treatment effect across trials with the random-effect model if I2 >50%, and with the fixed-effect model if I2 >50%.

## 3. Results

### 3.1. Description of Included Studies

The search produced 131 trials from all of the databases searched. Among these 131 studies, 49 either did not include Chinese herb, or were not randomised controlled trials, or were reviews, or did not report original data. Through reading the full text, 56 studies' level of evidence was graded scores lower than 3 according to the Jadad quality score. At the end, 12 published RCTs were included.  Table 3 shows baseline characteristics of the 12 studies.

### 3.2. Risk of Bias in Included Studies

**Figure 1:**
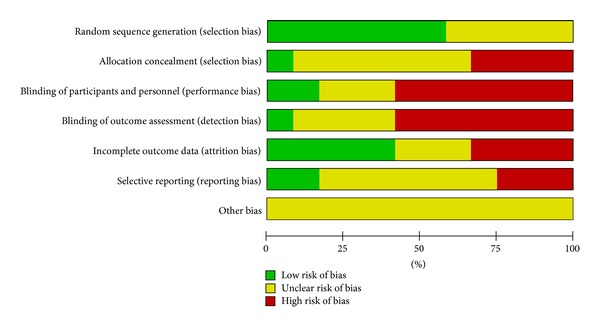
Risk of bias graph: review authors' judgments about each risk of bias item presented as percentages across all included studies.

**Figure 2:**
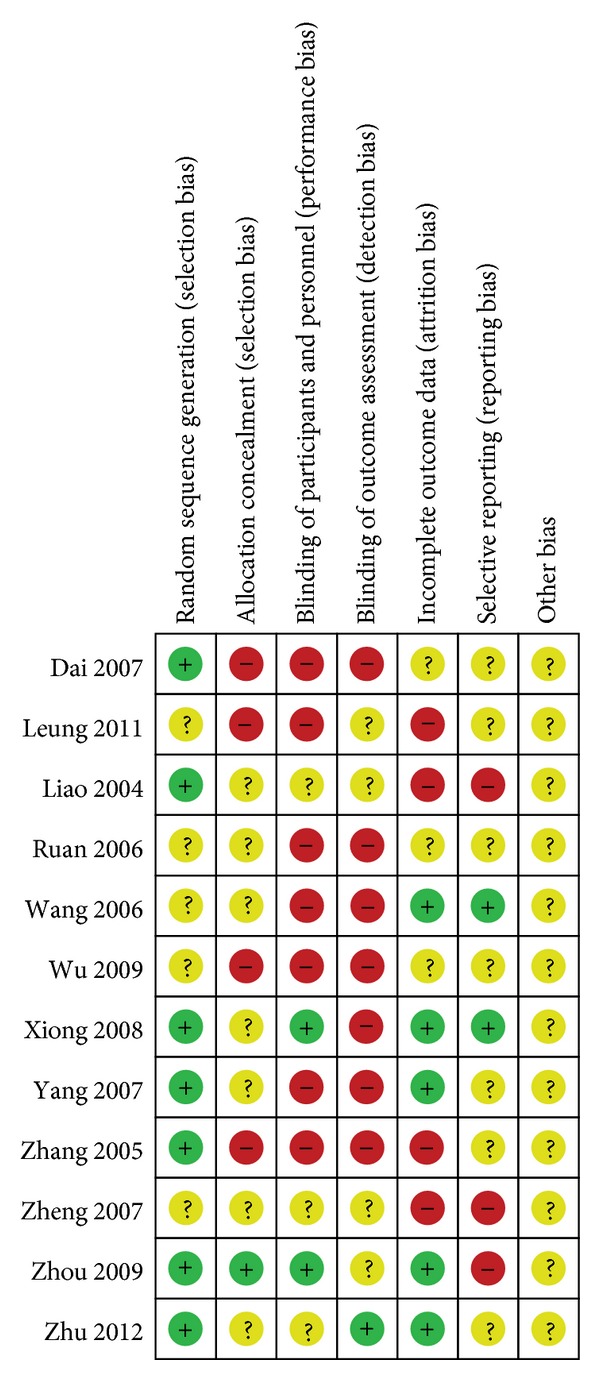
Risk of bias summary: review authors' judgments about each risk of bias item for each included study.

The reports of all trials mentioned randomization, but only seven described the method of randomization [10–16]. In addition, the reports of seven trials mentioned double blinding [11–13, 15, 17–19], and the report of one trial mentioned single blinding in their methodological design [14]. We also grade all included studies by (Table 2) which was recommended by Cochrane Handbook 5.0. Figures 1 and 2 are made to show the results of the author's judgment about each methodological quality item for each included studies. 

### 3.3. Effects of Interventions

#### 3.3.1. Effects of Chinese Herbs versus Placebo on Spine BMD

**Figure 3:**
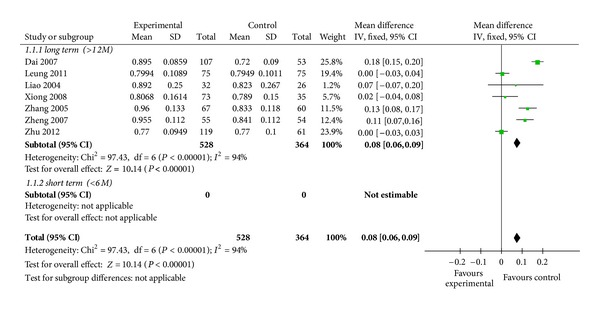
Chinese herbs versus placebo on spine BMD.

7 of all included RCTs studied effects of Chinese herbs versus placebo on lumbar spine areal BMD. The pooled data showed that the percent change of increased BMD in the spine is higher with Chinese herbs in comparison to treatment with placebo after 6 months (WMD = 0.07, 95% CI: 0.01–0.04, *n* = 892) (Figure 3).

#### 3.3.2. Effects of Chinese Herbs versus Placebo on Femoral Neck BMD

**Figure 4:**
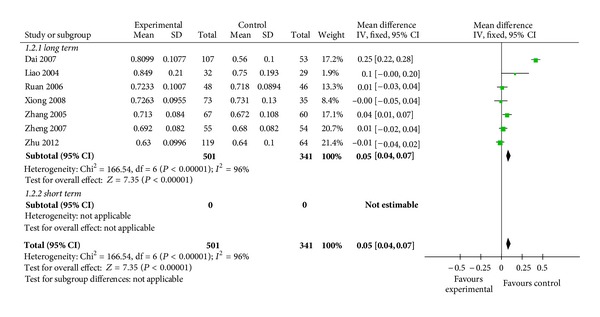
Chinese herbs versus placebo on femoral neck BMD.

7 RCTs reported the femoral neck BMD. In the femoral, Chinese herb showed no significant increments of BMD compared to placebo (WMD = 0.06, 95% CI: −0.02–0.13, *n* = 842) (Figure 4).

#### 3.3.3. Effects of Chinese Herbs versus Standard Antiosteoporotic Drugs on BMD

**Figure 5:**
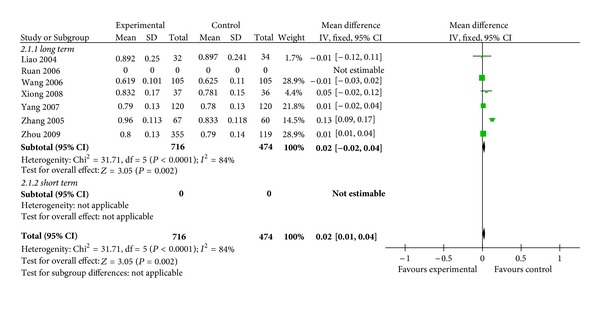
Chinese herbs versus standard anti-osteoporotic drugs on lumber spine BMD.

**Figure 6:**
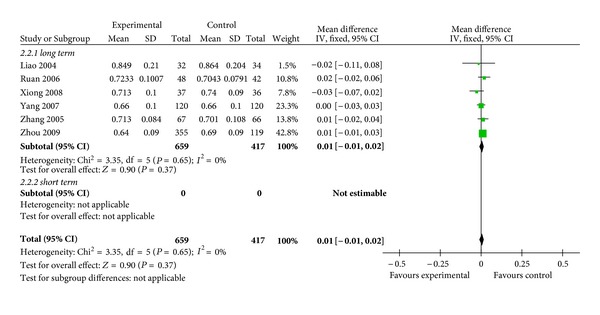
Chinese herbs versus standard anti-osteoporotic drugs on the femoral neck BMD.

To compare the efficacy of the Chinese herbs with the standard anti-osteoporotic drugs, a subgroup analysis has been made. 2 of the included RCTs used calcitriol for subjects in the control groups, 2 used HRT treatment for subjects in the control groups and 4 of all included RCTs used another phytotherapy as the control groups. Chinese herbs also show advantage in lumber spine BMD change (WMD = 0.03, 95% CI: −0.01–0.08, *n* = 1109) (Figure 5). In this pooled analysis, the increases in the femoral neck BMD values were not significant between Chinese herbs and standard anti-osteoporotic drugs treatments (WMD = 0.01, 95% CI: −0.01–0.02, *n* = 1076) (Figure 6).

## 4. Discussion

### 4.1. Summary of Main Results

Although there are many reports in China endorsing the therapeutic value of various herbs originally used as “Kidney tonics” against “Kidney deficiencies,” the claims have not been based on reliable clinical trials. This systematic review shows a trend for traditional Chinese medicine to increase spine BMD in Chinese patients with osteoporosis. Due to the data of 12 included trials (1816 patients involved), our analysis is strengthened with acceptable standard and methodology.

### 4.2. Overall Completeness and Applicability of Evidence

The reports of 4 of the 12 included trials provided data on the changes in lumbar and femoral BMD in anti-osteoporotic drugs treatment groups. The anti-osteoporotic drugs resulted in an increase of 0.0897 ± 0.0241 g/cm^2^ in lumber BMD and 0.0757 ± 0.0167 g/cm^2^ in femoral BMD compared with baseline values. The changes in BMD caused by the drugs in the included RCTs were in accordance with the findings of other RCTs conducted in different countries [19]. We systematically reviewed clinical trials and synthesized data from 12 trials in this study. The results showed similar pharmacological effects between Chinese herbs and standard anti-osteoporotic drugs in the regulation of bone turnover. The review indicated the potential of Chinese herbs for the treatment of osteoporosis. It is postulated that some effective components in these herbs were responsible for the anti-osteoporosis drugs like activities in clinical treatment. But there is still no specific target of pharmacological action to indicate that the efficacy would be inferior to target orientated pharmaceuticals. A longer period of observation and a large sample size might be mandatory for a more scientific revelation of the result of treatment.

### 4.3. Quality of the Evidence

The quality of the study designs and descriptions of the original trials are critical issues for meta-analyses. All of the 12 RCTs included in this study were assessed by the Jadad scale, and all the 12 studies were accepted as high quality trials. Although we included RCTs in high quality for data analysis in this study only, selection and detection biases would have existed if blinding had failed.

### 4.4. Potential Biases in the Review Process

Similar to other meta-analyses, our study has some limitations. First, the analysis is only based on published data, and no unpublished data are found. Second, differences in treatment length and design as well as in the severity of disease of the participants are also factors that potentially introduce difficulties in the analysis, as large differences in treatment effects can be expected. Moreover, the presented analysis was not designed to assess incident fractures. Finally, we suggest a longer duration of future studies as a 6-month study may not be long enough to reveal BMD changes, especially when the sample size is relatively small.

### 4.5. Potential Mechanism of Action

Chinese herbal medicine (CHM), a pharmaceutical part of TCM, has a long history of use, with extensive literature and clinical applications covering thousands of years. Chinese herbal formulae lack a well-defined mechanism of action. Plants are rich in a variety of compounds. It may therefore be necessary to identify the active ingredient(s) from a herbal extract for mechanistic investigations [22, 23]. Up till now, many active ingredients have been isolated from commonly used CHM [24].

The chemical composition of naturally grown herb may vary according to climatic conditions, harvest time, storage condition, and so on. These variabilities can result in significant differences in pharmacological activity, making standardization of botanical difficult [25]. At the present time, there are 15 major categories of active ingredients in CHM, including flavones, alkaloids, glucides, glycosides, volatile oils, resins, phytochromes, organic acids, amino acids, tannins, proteins, enzymes, trace elements, polysaccharides, and mineral salts [23].

Compounds that are identified by activity-guided fractionation must be tested in appropriate animal models to confirm in vivo activity [23, 26]. 

Curculigo orchioides (CO), which belongs to the Amaryllidaceae family, is mainly distributed in the subtropical regions of Asia, especially in southern China and India. Previous phytochemical investigations into rhizomes of this species revealed the presence of phenols and phenolic glycosides, including curculigoside, curculigoside B, curculigoside C, the triterpene saponins curculigosaponins A–M, and other compounds, including 1,3,7-trimethylxanthine, daucosterol, and aliphatic long-chain ketones [27, 28], of which phenols and phenolic glycosides have potential antioxidant activities [29, 30] and immunostimulatory effects [31, 32]. Administration of CO extract prevented bone loss in the trabecular bone of the tibia in ovariectomized rats without affecting the weight of the body and the uterus, increased serum phosphorus, calcium, and OPG levels, and decreased serum DPD/Cr, TRAP, ACTH, and corticosterone levels, but did not alter serum TNF-1, IL-6, and ALP levels in ovariectomized rats. CO ethanol extract has a definite protective effect on bone loss in ovariectomized rats by inhibiting bone resorption and increasing serum phosphorus and calcium levels, without affecting bone formation [33]. In traditional Chinese medicine, CO rhizomes are considered to have the effects of maintaining healthy energy and nourishing the liver and kidneys [34, 35], and are thus widely used to treat diseases and disorders of bone metabolism. In most Chinese herbal formulae, they are also used to treat osteoporosis [36]. Herbaepimedii which belongs to the Berberidaceae family, contains a plenty of Isoflavone [37]. Isoflavone is also one of the determined substance in most of the recipes. Pharmacological studies, either on murine models of osteoporosis or in vitro, have provided some convincing evidence of positive effects of soya and isoflavones on bone health [38, 39]. Rutin, the glycosylated form of quercetin, is abundant in onion. It was proposed that at 200–600 mg/kg it was the pharmacologically active compound, although few studies have confirmed this. The vitro trails showed that rutin consumption increased femoral strength and trabecular bone density by decreasing bone resorption, although cortical bone density was unchanged. Rats supplemented with rutin also had higher plasma osteocalcin concentrations, indicating an increase in bone formation [40]. Cistanche salsa or the Chineses name of Rou Cong Rong, has been shown to have antiosteoporotic effects in ovariectomized mice. It has been reported by Yamaguchi et al. [41] that 16 mg/day of C. salsa significantly suppressed the femoral bone weight loss caused by ovariectomy; in fact femoral weight increased to 109% of the control in this study. Subsequently, the active compound was isolated and the structure elucidated as (2E,6R)-8-hydroxy-2,6-dimethyl-2-octenoic acid, a novel monoterpene which was found to be antiosteoporotic. This compound significantly suppressed femoral bone loss in ovariectomized mice at a concentration of 1.6–8 *μ*g/kg. It was concluded that the mechanism of action was different to that of antiosteoporotic agents such as 17*β*-estradiol since uterine weight was not affected. Drynariae rhizoma is a major component in 56 of 73 fracture prescriptions in traditional Chinese and is also used in Korean medicine. Its effects on protease activity involved in the initiation of bone loss in rats and mice were tested [42]. Both ethanol and aqueous extracts were potent inhibitors of cathepsins K and L, which denature the collagen in bone, with the ethanol extract being more potent. Later a study showed the beneficial effects of D. rhizoma on the proliferation of human bone cells, and immunomodulatory activity in vitro [43]. Human osteoprecursor cells (OPC-1) were cultured with differing concentrations of D. rhizoma and their proliferation was studied. Concentration of ≤120 *μ*g/mL enhanced proliferation, whereas >250 *μ*g/mL was inhibitory. Recent in vivo work has confirmed these results [44]. A study investigated Puerariae radix effects on bone loss in castrated mice, as it had previously been shown to exhibit an effect in ovariectomized mice. Male mice were given a dose of either different concentrations in the diet, and another group were given 17*β*-estradiol at a dose of 0.03 *μ*g/day for comparison. It was shown that even at low doses. Puerariae radix reversed the bone loss induced by castration, with femur BMD and trabecular number increasing, and trabecular separation decreasing [45]. Onobrychis ebenoides is well known as having oestrogenic activity in vitro. This, along with its bone-sparing effect, was described in a study. OVX rats were given 300 mg/kg body weight of extract of OE every day for 6 months which resulted in an increase of tibial BMD from that of the OVX control [46]. Du-Zhong cortex extract, which is rich in polyphenolic compounds such as lignans, phenolic acid, and flavonoids. Dose-dependently inhibited total BMD decrease in the femur caused by OVX, which was accompanied by a significant decrease in skeletal remodeling, as was evidenced by the decreased levels of the bone turnover markers osteocalcin (OC), alkaline phosphatase (ALP), deoxypyridinoline (DPD), and urinary Ca and P excretions. Analysis of the femoral metaphysis showed that DZCE at the highest doses (500 mg/kg/day) significantly prevents decrease in bone volume/tissue volume (BV/TV), connect density (Conn.D), trabecula number (Tb.N), and trabecula thickness (Tb.Th), and increase in trabecula separation (Tb.Sp) and structure model index (SMI) in OVX rats [47]. Treatment of OVX rats with Fructus Ligustri Lucidi extract could prevent OVX-induced increase in bone turnover by suppression of both serum osteocalcin and urinary deoxypyridinoline levels. In addition, Fructus Ligustri Lucidi extract could prevent OVX-induced loss of calcium in rats by increasing the intestinal calcium absorption rate, suppressing urinary Ca excretion as well as increasing bone calcium content [48]. Obvious separation trend between control and model group was found in principal component analysis score plot, the anti-osteoporosis effect of Rhizoma Drynariae can be indicated in partial least squares discriminant analysis score plot among these three groups. Six potential metabolite biomarkers, lysophosphatidylcholines, tryptophane, and phenylalanine, which were proved to be related with osteoporosis, were identified in the rats plasma. Compared with control group, level of all biomarkers increased significantly in model group, while that was much closer to normal in treatment group [49]. Achyranthes bidentata Blume is rich in active phytochemical compounds such as saponins, ketosteroids and flavonoids. The study demonstrated that five new oleanolic acid glycosides from Achyranthes bidentata could inhibit the formation of osteoclast, ecdysterone from Achyranthes bidentata increased osteoblastic activity, and flavonoid quercetin decreased osteoclastic differentiation. 16 weeks treatment of Achyranthes bidentata root extract slowed down the body weight gain and prevented the loss of bone mass induced by the OVX. The prevention effect on bone loss was due to altering the rate of bone remodeling, which could be inferred from the decreased level of bone turnover markers, such as serum alkaline phosphatase (ALP), osteocalcin (OC), and urinary deoxypyridinoline (DPD). The changes of urinary calcium and phosphorus excretion provided the same evidence. The treatment could also enhance the bone strength and prevent the deterioration of trabecular microarchitecture [50]. Cibotium barometz is a kind of usual herbs to treat osteoporosis, its extract prevented total BMD decrease in the femur induced by OVX, which was accompanied by a significant decrease in skeletal remodeling, as was evidenced by the decreased levels of the bone turnover markers, such as osteocalcin (OC), alkaline phosphatase (ALP), deoxypyridinoline (DPD), and urinary Ca and P excretions. The treatment could also enhance the bone strength and prevent the deterioration of trabecular microarchitecture [51]. 

## 5. Conclusions

We conclude that Chinese herbs substantially increased BMD of the lumbar spine compared to placebo or anti-osteoporotic drugs as indicated in the current clinical reports on osteoporosis treatment. Long term of Chinese herbs over 12 months of treatment duration may increase BMD in the hip more effectively. However, further studies are needed to corroborate the positive effect of increasing the duration of Chinese herbs on outcome as the results in this analysis are based on indirect comparisons. To date there are no studies available that compare Chinese herbs, Chinese herbs plus anti-osteoporotic drugs, and anti-osteoporotic drug versus placebo in a factorial design. Consequently, we are unable to draw any conclusions on the possible superiority of Chinese herbs plus anti-osteoporotic drug versus anti-osteoporotic drug or Chinese herb alone in the context of BMD.

## Figures and Tables

**Table 1:** Assessing the quality of reports of randomized clinical trials.

Item	Score
Randomization	
Was the study described as randomized (this includes the use of words such as randomly, random, and randomization)?	1
If the first answer is yes, the method to generate the sequence of randomization was described and it was appropriate (table of random numbers, computer generated, etc.)	1
If the first answer is yes, the method to generate the sequence of randomization was described and it was inappropriate (patients were allocated alternately, or according to date of birth, hospital number, etc.)	−1

Double blind	
Was the study described as double blind?	1
If the first answer is yes, the method of double blinding was described and it was appropriate (identical placebo, active placebo, dummy, etc.)	1
If the first answer is yes, the method of blinding was inappropriate (e.g., comparison of tablet versus injection with no double dummy)	−1

Description of withdrawals and dropouts?	
Was there a description of withdrawals and dropouts?	1

**Table 2:** The Cochrane Collaboration's tool for assessing risk of bias.

Random sequence generation	
Low risk of bias	The investigators describe a random component in the sequence generation process such as: referring to a random number table; using a computer random number generator.
High risk of bias	The investigators describe a nonrandom component in the sequence generation process. Usually, the description would involve some systematic, nonrandom approach, for example, sequence generated by odd or even date of birth; sequence generated by some rule based on date (or day) of admission.
Unclear risk of bias	Insufficient information about the sequence generation process to permit judgement of “Low risk” or “High risk.”

Allocation concealment	

Low risk of bias	Participants and investigators enrolling participants could not foresee assignment because one of the following, or an equivalent method, was used to conceal allocation: central allocation (including telephone, web-based and pharmacy-controlled randomization); sequentially numbered drug containers of identical appearance.
High risk of bias	Participants or investigators enrolling participants could possibly foresee assignments and thus introduce selection bias, such as allocation based on using an open random allocation schedule (e.g., a list of random numbers); assignment envelopes were used without appropriate safeguards (e.g., if envelopes were unsealed or nonopaque or not sequentially numbered).

Blinding of participants and personnel	

Low risk of bias	Any one of the following: no blinding or incomplete blinding, but the review authors judge that the outcome is not likely to be influenced by lack of blinding; blinding of participants and key study personnel ensured, and unlikely that the blinding could have been broken.
High risk of bias	No blinding or incomplete blinding, and the outcome is likely to be influenced by lack of blinding; blinding of key study participants and personnel attempted, but likely that the blinding could have been broken, and the outcome is likely to be influenced by lack of blinding.
Unclear risk of bias	Any one of the following: insufficient information to permit judgement of “Low risk” or “High risk”; the study did not address this outcome.

Blinding of outcome assessment	

Low risk of bias	Any one of the following: no blinding of outcome assessment, but the review authors judge that the outcome measurement is not likely to be influenced by lack of blinding; blinding of outcome assessment ensured, and unlikely that the blinding could have been broken.
High risk of bias	Any one of the following: no blinding of outcome assessment, and the outcome measurement is likely to be influenced by lack of blinding; blinding of outcome assessment, but likely that the blinding could have been broken, and the outcome measurement is likely to be influenced by lack of blinding.
Unclear risk of bias	Any one of the following: insufficient information to permit judgement of “Low risk” or “High risk”; the study did not address this outcome.

Incomplete outcome data	

Low risk of bias	Any one of the following: no missing outcome data; reasons for missing outcome data unlikely to be related to true outcome (for survival data, censoring unlikely to be introducing bias).
High risk of bias	Any one of the following: reason for missing outcome data likely to be related to true outcome, with either imbalance in numbers or reasons for missing data across intervention groups; for dichotomous outcome data, the proportion of missing outcomes compared with observed event risk enough to induce clinically relevant bias in intervention effect estimate.
Unclear risk of bias	Any one of the following: insufficient reporting of attrition/exclusions to permit judgement of “Low risk” or “High risk” (e.g., number randomized not stated, no reasons for missing data provided); the study did not address this outcome.

Selective reporting	

Low risk of bias	Any of the following: the study protocol is available and all of the study's pre-specified (primary and secondary) outcomes that are of interest in the review have been reported in the prespecified way; the study protocol is not available but it is clear that the published reports include all expected outcomes, including those that were pre-specified (convincing text of this nature may be uncommon).
High risk of bias	Any one of the following: not all of the study's prespecified primary outcomes have been reported; one or more primary outcomes is reported using measurements, analysis methods, or subsets of the data (e.g., subscales) that were not prespecified.
Unclear risk of bias	Insufficient information to permit judgement of “Low risk” or “High risk.” It is likely that the majority of studies will fall into this category.

Other bias	

Low risk of bias	The study appears to be free of other sources of bias.
High risk of bias	There is at least one important risk of bias. For example, the study had a potential source of bias related to the specific study design used, or has been claimed to have been fraudulent; or had some other problem.
Unclear risk of bias	There may be a risk of bias, but there is either insufficient information to assess whether an important risk of bias exists or insufficient rationale or evidence that an identified problem will introduce bias.

**Table 3:** Characteristics of clinical trials of Chinese herbs for osteoporosis.

Trial year prevention/treatment (reference)	Number of patients (treatment/control)	Intervention	Duration	Outcomes measured
Dai and Shen 2007 [10]	107 versus 85	MGT 1.5 g/day∖XLGB 1.5 g/day versus placebo, all: (calcium 1000 mg/day)	6 M	BMD: lumber spine and femoral neck
Leung et al. 2011 [17]	75 versus 75	ELP 2.28 g/day versus placebo	12 M	BMD: lumber spine and femoral neck
Wu et al. 2009 [20]	25 versus 25	XLGB 1.5 g/day versus Calcitriol 0.25 *μ*g + calcium 700 mg/day + vitamin D400 IU/day	6 M	BMD: lumber spine and femoral neck
Zhang et al. 2005 [11]	67 versus 66 versus 60	YGC 120 g/day versus Calcitriol 0.25 *μ*g versus placebo, all: (calcium 510 mg/day)	6 M	BMD: lumber spine and femoral neck
Ruan et al. 2006 [21]	48 versus 42	QGJN 0.75 g/day versus oral estradiol valerate 0.5–1.5 mg/day	6 M	BMD: lumber spine
Zhu et al. 2012 [12]	109 versus 61	XLGB 6 g/day versus placebo	12 M	BMD: lumber spine and femoral neck
Xiong et al. 2008 [13]	73 versus 35	JGKL 10 g/day versus placebo, all: (calcium 510 mg/day)	6 M	BMD: lumber spine and femoral neck
Zhou et al. 2009 [14]	355 versus 119	MGT 1.5 g/day versus XLGB 1.5 g/day	6 M	BMD: lumber spine and femoral
Liao et al. 2004 [15]	32 versus 34	BSSS soup 200 mL/day versus oral conjugated estrogen 0.5 mg/d and medroxyprogesterone 2.5 mg/d	6 M	BMD: lumber spine and femoral
Yang et al. 2007 [16]	120 versus 120	XLGB 1.5 g/day versus GKKFY 20 mL/day	6 M	BMD: lumber spine and femoral neck
Wang et al. 2006 [18]	105 versus 105	GSKC 2.56 g/day versus GSKT 20 g/day	6 M	BMD: femoral neck
Zheng et al. 2007 [19]	55 versus 54	JWGT 3 g/day versus placebo, all: (calcium 510 mg/day)	6 M	BMD: lumber spine and hip

MGT: Migu tablet; XLGB: Xian ling Gubao capsule; ELP: Bo-gu Ling capsules; YGC: Yigu capsule; QGJN: Qiang-Gu capsule; JGKL: Jiangu granule; BSSS: Bu Shen Sheng Sui soup; GKKFY: Gu Kang Oral liquid; GSKC: Gushukang capsule; GSKT: Gushukang granules; JWGT: Jinwugutong capsule; BMD: bone mineral density.
